# Cyanobacteria and microalgae bioactive compounds in skin-ageing: potential to restore extracellular matrix filling and overcome hyperpigmentation

**DOI:** 10.1080/14756366.2021.1960830

**Published:** 2021-08-06

**Authors:** Rita Favas, Janaína Morone, Rosário Martins, Vitor Vasconcelos, Graciliana Lopes

**Affiliations:** a CIIMAR/CIMAR, Interdisciplinary Centre of Marine and Environmental Research, Matosinhos, Portugal; b FCUP, Department of Biology, Faculty of Sciences, University of Porto, Porto, Portugal; c Health and Environment Research Centre, School of Health, Polytechnic Institute of Porto, Porto, Portugal

**Keywords:** Skin ageing, matrix metalloproteinases, collagenase, elastase, hyaluronidase, hyperpigmentation

## Abstract

As the largest organ in human body, skin acts as a physicochemical barrier, offering protection against harmful environmental stressors, such as chemicals, pathogens, temperature and radiation. Nonetheless, skins prominence goes further, with a significant psychosocial role in an increasingly ageing population. Prompted by consumers’ concern regarding skincare, cosmetic industry has been developing new formulas capable of lessening the most visible signs of ageing, including reduction in skin density and elasticity, wrinkling and hyperpigmentation. Allied to skincare is the rising importance set on natural products, sustainably obtained from less environmental impacting methods. Cyanobacteria and microalgae are adding importance in this field, given their ability to biosynthesize secondary metabolites with anti-ageing potential. In this review, we present an overview on the potential of cyanobacteria and microalgae compounds to overcome skin-ageing, essentially by exploring their effects on the metalloproteinases collagenase, elastase, gelatinase and hyaluronidase, and in other enzymes involved in the pigmentation process.

## 1. Introduction

**Figure 1.**
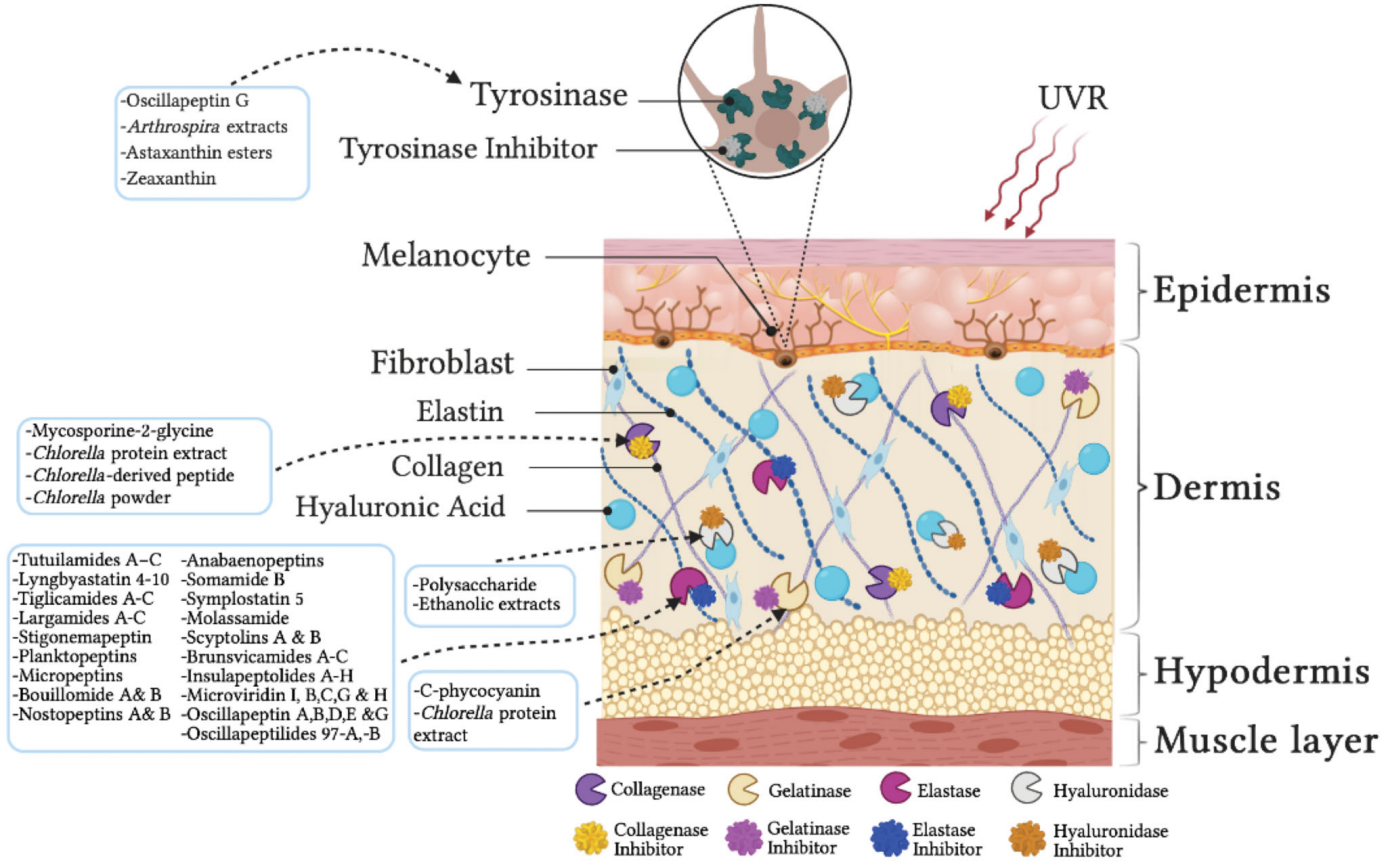
Schematic representation of skin structure, with emphasis on the main targets of cyanobacteria and microalgae bioactive compounds. UVR: ultraviolet radiation. *Created with BioRender.com*.

Representing 16% of the total body weight, skin is the largest human organ. Among its several functions, skin works as a physical barrier, offering protection against harmful stressors, such as chemicals, pathogens, cold, heat and ultraviolet radiation (UVR)^1^. In addition, skin plays a crucial role in the synthesis of vitamin D, essential to the maintenance of calcium homeostasis, as well as in immune, sensorial and body temperature regulation functions^2^^,^^3^. Structurally, skin is composed of three distinct layers: epidermis, dermis and hypodermis^4^ (Figure 1). The most superficial and exposed layer, epidermis, is a continuous renewing stratified keratinised squamous epithelium, constituted mainly by keratinocytes and melanocytes. Its primary function relies on protection against environmental chemical and biochemical threats, functioning as a physical and adaptive immunologic barrier^5^. Underlying the epidermis, there is dermis, constituted by connective tissue that includes an extracellular matrix (ECM) and cells like fibroblasts and macrophages. ECM is a three-dimensional network of collagen and elastin fibres surrounded by the ground substances, such as hyaluronic acid (HA), acting together to maintain skin filling, elasticity and flexibility^4^ (Figure 1). Any imbalance between these main components may result in the loss of skin structure, leading to an unhealthy and aged appearance^4^. Given its crucial role in personal feature and social welfare, the preservation of all skin layers has become one of the main requirements of modern societies, which has driven the development of new and innovative products by pharmaceutical and cosmetic industries^6^.

Skin care and beauty products have played important roles in human history^7^. The oldest records on cosmetics came from Egyptians, who were particularly concerned with physical appearance, namely with the development of facial wrinkles. Due to the dry and hot weather to which population was exposed, the skin care with the use of oils and creams were part of the daily routine^8^. Over the years, other products like salts, honey and hydroxy and tartaric-acids were also used for skin treatment and cleaning. With origin in the ancient Roman public baths, the term “*cosmetic*”, meaning to “beautify the body”, came up^9^. Currently, cosmetic products are defined by the European Commission (EC) regulation No 1223/2009 as “*any substance or mixture intended to be placed in contact with the external parts of the human body (epidermis, hair , nails, lips and external genital organs) or with teeth and the mucous membranes of the oral cavity, with a view to clean, perfume, change the appearance, protect, keep in good condition or correct body odours*”^10^. Globally, the cosmetic industry has been one of the least impacted from the oscillation of the financial markets. According to a very recent survey^11^, it is predicted an economic volume of $805.61 billion by 2023, as a result of an increase in global consumption. Likewise, the rise in average life expectancy has led to a robust demand for anti-ageing products, thus creating room for countless innovations and boosting the industry growth^6^.

A main society demand concerning skin is in fact the delay of skin-ageing. This slow and complex process is induced by endogenous factors such as genetics, and exogenous factors such as personal habits and environment^12^. Endogenous ageing is a natural process where skin gradually loses its functional and structural characteristics, as a natural consequence of cellular senescence due to a decrease in cellular metabolism, DNA repair capability, gene mutations, loss of telomeres, chromosomal abnormalities, and hormonal changes^13^^,^^12^. On the other hand, exogenous ageing is caused by chemicals, toxins, pollutants, extreme conditions of cold or heat and radiation^14^. In both cases, the phenomenon commonly affects the epidermal thickness, structure and pigmentation, as well as the morphology and microstructure of the deeper layers^15^, resulting in thinning, dryness, flaccidity, enlarged pores, fine lines and wrinkles, dark spots and hyperpigmentation^14^.

In the last decades, scientific research underwent a significant evolution in the field of anti-ageing products, with focus on natural sources and green processes, free of animal testing and with a green life cycle, including packaging, manufacturing, distribution, post-consumer use, and sourcing^16^. As a result, a substantial expansion of cosmetic industries emerged and a countless number of new products have been launched to the market^6^. While plants have been the primary raw material for cosmetics production for centuries, the exhaustion in this over-studied resource led to the use of other organisms such as macroalgae and eukaryotic microalgae, namely from marine origin^17^. Marine organisms have thus emerged as a prolific source of cosmetic ingredients able to minimise damages that occur during skin ageing, such as the formation and exacerbation of wrinkles^4^, pigmentation, collagen degradation and loss of elasticity^18^ and loss of moisture^19^. Among them, cyanobacteria have gained importance, due to their capacity to produce bioactive secondary metabolites, with unique structures and mechanisms of action. These gram-negative bacteria represent the only group of prokaryotes that can perform oxygenic photosynthesis, similarly to plants, although with a higher photosynthetic rate and biomass production^20^^,^^21^. Their capacity to self-renew, basic nutritional requirements^22^, minimal cultivation space and low environmental impact^17^^,^^23^, makes them a sustainable choice for skin care products. Their residual biomass can be used as fertiliser or in animal feed, and can generate bio-polyesters, known as “Green Plastics”, thus fitting the concept of circular economy^17^^,^^24^^,^^25^. Given this, marine organisms, and particularly, microorganisms, can be seen as a new hope in the search for new and innovative bioactive molecules, able to counteract the reactions leading to skin damage and ageing.

## 2. Methods

The aim of this review was to compile the available studies on extracts or bioactive compounds produced by cyanobacteria and microalgae to potentially restore the skin ECM and overcome hyperpigmentation. The review was conducted using Scopus, Web of Science, PubMed, ScienceDirect, ResearchGate, and Google Scholar databases. Query terms included "cyanobacteria", "microalgae", "bioactive compounds", "skin-ageing", "metalloproteinases", “collagenase”, “gelatinase”, “elastase”, “hyaluronidase”,“tyrosinase” and "hyperpigmentation". In addition, we have supplemented the search by further exploring references of the articles retrieved from the referred databases.

## 3. Cyanobacteria and microalgae in skin-ageing

Cyanobacteria and microalgae are prolific sources of natural bioactive compounds with different areas of application^22^. It is known that cyanobacteria and microalgae synthesise pigments, lipids (polyunsaturated fatty acids - PUFAs, hydrocarbons), proteins, polysaccharides (cellulose, alginates, starch), and other compounds, with proven bioactivities in the pharmaceutical, energy, nutrition and cosmetic fields^24^^,^^26^^,^^27^. In relation to energy application, diverse microalgae are being used to produce bioethanol, biogas, and biohydrogen. Due to its high protein and PUFAs content, they can also be used for human and animal nutrition^24^. In the pharmaceutical field, it is noteworthy the production of grassystatin A–B for lung cancer, kempopeptin A for colon cancer, and dolastatin 15 for breast cancer^28^. Other studies show that they also have antitumor, anticoagulant, anti-inflammatory, and protease inhibitory activities^28^. Regarding cosmetics, their bioactive compounds, mostly as extracts, have been reported to be used in shampoos, and body soaps^10^^,^^19^, face lotions, anti-ageing creams, makeup and sun blockers^17^^,^^19^^,^^22^. Concerning sunscreens, some of these microorganisms produce UV-absorbing compounds, such as mycosporine-like amino acids (MAAs) and scytonemin, as well as carotenoids, phycobiliproteins and polyphenols, with an important role in preventing oxidative stress through their capacity to scavenge deleterious free radicals^29^. They also produce exopolysaccharides (EPS), with important moisturising properties^17^, metalloproteinase inhibitors^30^, and compounds able to inhibit tyrosinase, and thus avoid skin hyperpigmentation^17^.

### 3.1. ECM-target compounds

The dermis is constituted by loose and dense connective tissue in which ECM constitutes the major component. ECM is a gel-like material made of collagen and elastic fibres dispersed in a ground substance made of glycosaminoglycans, proteoglycans, and connective tissue glycoproteins. It is essential to hold cells together, and to provide a pathway for nutrients and oxygen to the epidermis^31^. Several cell types, such as keratinocytes, fibroblasts, macrophages, endothelial cells, mast cells, eosinophils and neutrophils, are capable of producing specific enzymes responsible for the ECM turnover and, in some situations, leading to the loss of skin structure and appearance of wrinkles^4^. Recently, there has been more research on metalloproteinases, and in their effect on the dermal matrix structure, as well as in enzymes responsible for skin pigmentation. Both metalloproteinases and skin pigmentation-associated enzymes have become targets for bioactive compounds with anti-ageing potential. Hence, we present below an overview on the potentialities of cyanobacteria and microalgae-derived compounds to overcome skin-ageing, focussing the main enzymes responsible for the maintenance of dermal matrix structure.

#### 3.1.1. Metalloproteinases

Matrix metalloproteinases (MMPs) are a family of extracellular zinc-dependent enzymes, which main function is to remodel and degrade the ECM^30^. Collagen and elastin are primary proteins of the ECM, responsible for resistance and elasticity of the skin^32^. Therefore, any alterations in collagen and elastin induced by MMPs, will contribute to the loss of dermal structure, resulting in its damage^33^. A main skin stress condition is the exposition to UVR, that exacerbates the degradation of the ECM collagen and elastin fibres through the induction of MMPs activity^4^. Although MMPs are crucial to epidermal differentiation and prevention of wound scars, their up-regulation potentiates the signs of ageing and the development of skin cancer^30^.

Despite the existence of different subgroups of MMPs, such as collagenases, gelatinases, stromelysins, matrilysins, membrane-type MMPs (MT-MMPs), among others^34^, this review will focus on the most relevant regarding skin ageing: collagenase, gelatinase, elastase, and hyaluronidase (Figure 1).

##### 3.1.1.1. Collagenases

There are different subtypes of collagenases, e.g. MMPs-1, -8, -13, and -18, which are proteolytic enzymes responsible for the initiation of collagen fragmentation in human skin, and for the control of collagen turnover^35^. These enzymes cleave all types of interstitial collagens in the skin (I, II, and III) at a single site. After cleavage, the collagen fragments lose stability at body temperature and their structure is disrupted, contributing to the loss of dermal homeostasis and leading to tissue damage^30^^,^^36^^,^^37^. Hence, inhibiting MMPs constitutes a strategy to conserve the dermal matrix structure, avoiding tissues damage and delaying the formation of wrinkles.

**Figure 2.**
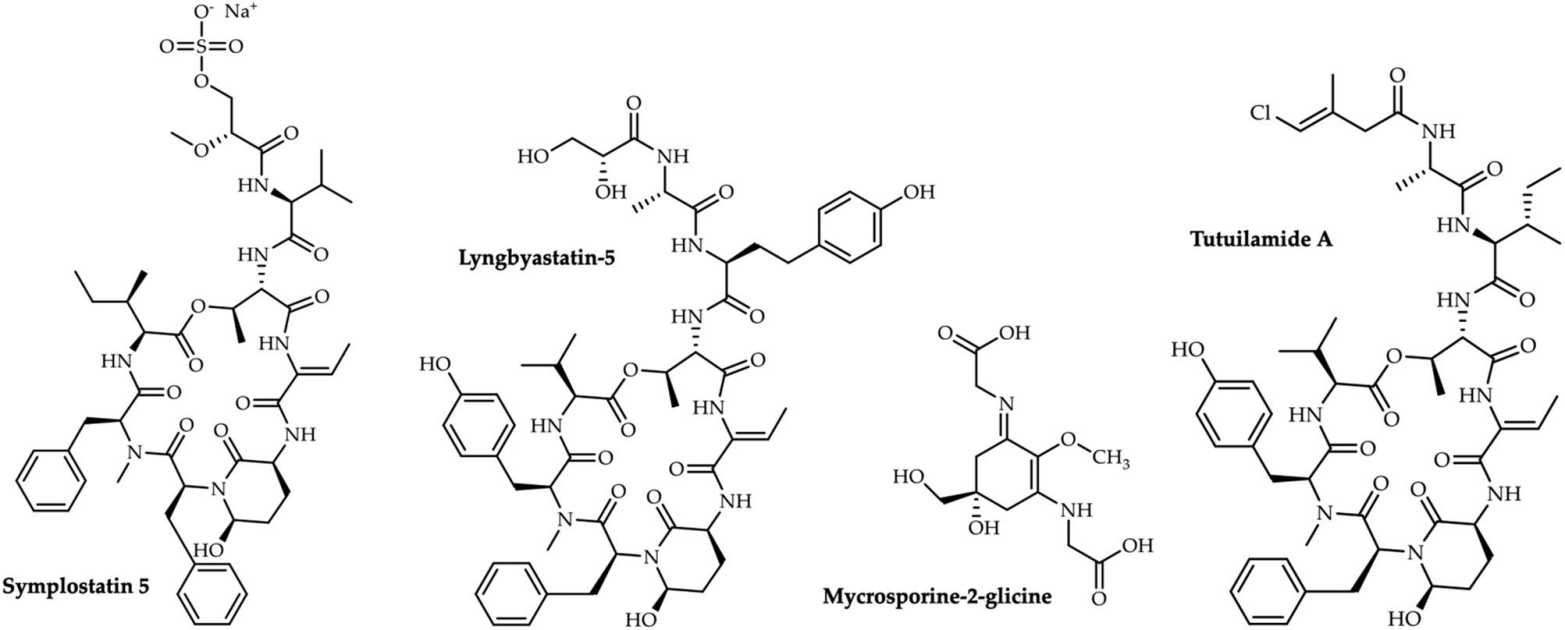
Structures of compounds isolated from cyanobacteria, with ability to inhibit matrix metalloproteinases.

Several recent reports point different compounds isolated from cyanobacteria and microalgae as potent inhibitors of enzymes responsible for the digestion of ECM components, essential to maintain dermal filling, and that are naturally decreased during the ageing process and exposition to deleterious abiotic factors^38^ (Figure 1, Table 1). An example is the mycosporine-2-glycine (M2G) (Figure 2), isolated from the cyanobacterium *Aphanothece halophytica*, that presented collagenase inhibitory properties, with a robust IC_50_ of 0.47 mmol/L, comparable to that of the well-known collagenase inhibitor phenanthroline. It was suggested that the mechanism of enzyme inhibition could be related to the capacity of M2G to chelate calcium ions, and to the efficiency of the compound in inhibiting the formation of glycation-dependent protein-protein cross-linking, a process associated to the development of dull skin and to the decrease in skin elasticity. These results have demonstrated M2G as an alluring candidate for the development of new anti-ageing cosmetics and emphasised its potential in the prevention of skin ageing^39^.

**Table 1.** Bioactive potential of cyanobacteria and microalgae-derived compounds against matrix metalloproteinases.^a^

MMP	Compound/extract	Species	Model	Reference
Collagenase	Mycosporine-2-glycine	*Aphanothece* *halophytica*	Collagenase from *Clostridium histolyticum*	Tarasuntisuk et al.^39^
	Protein extract	*Chlorella minutissima*	Human breast cancer cell line MDA-MB231	Kunte and Desai^40^
	*Chlorella*-derived peptide	*Chlorella* sp.	Human skin fibroblasts 966SK (BCRC 60153)	Chen et al.^41^
	*Chlorella* powder	*Chlorella pyrenoidosa*	Human MMP-1	Cheng et al.^97^
	*Arthrospira* -derived peptide	*Arthrospira maxima*	Collagenase from *Clostridium histolyticum*	Montalvo et al.^42^
	*Arthrospira* crude protein (SPCP)	*Arthrospira platensis*	Human dermal fibroblast cell line (CCD-986sk)	Liu et al.^98^
Gelatinase	C-phycocyanin extract	*Spirulina platensis*	HepG2 cell line	Kunte and Desai ^44^
	Protein extract	*Chlorella minutissima*	HepG2 cell line	Kunte and Desai ^40^
Elastase	Tutuilamides A − C	*Schizothrix* sp. *Coleofasciculus* sp.	PPE	Keller et al.^46^
	Lyngbyastatin 4 Lyngbyastatin 5 Lyngbyastatin 6 Lyngbyastatin 7 Cyclodepsipeptide somamide B Tiglicamides A-C Largamides A-C	*Lyngbya confervoides*	PPE	Matthew et al.^47^ Taori et al.^48^ Matthew et al.^50^ Matthew et al.^49^
	Lyngbyastatin 8 Lyngbyastatin 9 Lyngbyastatin 10	*Lyngbya semiplena*	PPE	Kwan et al.^51^
	Bouillomide A Bouillomide B	*Lyngbyabouillonii*	PPE	Rubio et al.^52^
	Symplostatin 5-10	*Symploca* sp.	PPE Human neutrophil elastase	Salvador et al.^54^
	Stigonemapeptin	*Stigonema* sp.	PPE	Kang et al.^53^
	Oscillapeptins A, B, D, E Oscillapeptin G Oscillapeptilides 97-A and -B Microviridin I	*Oscillatoria agardhii*	PPE	Itou et al.^55^ Fujii et al.^56^
	Microviridins G and H Nostopeptins A and B	*Nostoc minutum*	PPE	Murakami et al.^57^ Okino et al.^58^
	Microviridins B and C Micropeptins HH978, HH960, HH992, and DR1006	*Microcystis aeruginosa*	PPE	Okino et al.^59^ Lodin-Friedman and Carmeli^60^ Adiv et al.^61^
	Molassamide	*Dichothrix utahensis*	PPE	Gunasekera et al.^62^
	Scyptolins A and B	*Scytonema hofmanni*	PPE	Matern et al.^63^
	Planktopeptins BL1125, BL843, and BL1061 Anabaenopeptins B and F	*Planktothrix rubescens*	HLE/PPE HLE/PPE	Grach-Pogrebinsky et al.^64^ Bubik et al.^65^
	Brunsvicamides A-C	*Tychonema* sp.	HLE	Sisay et al.^66^
	Insulapeptolides A-H	*Nostoc insulare*	HLE	Mehner et al.^67^
	*Arthrospira* crude protein (SPCP)	*Arthrospira platensis*	Human dermal fibroblast cell line (CCD-986sk)	Liu et al.^98^
Hyaluronidase	Polysaccharide	*Nostochopsis lobatus* MAC0804NAN	Hyaluronidase from bovine testes, type IV-S	Yamaguchi and Koketsu et al.^75^
	Ethanolic extracts	*Spirulina platensis* *Chlorella pyrenoidosa* *Porphyridium purpureum* *Rhodosorus marinus* *Dunaliella salina* *Pleurochrysis carterae*	Hyaluronidase from bovine testes, type IV-S	Fujitani et al.^76^
	*Arthrospira*-derived peptide	*Arthrospira maxima*	Hyaluronidase from bovine testes, type IV-S	Montalvo et al.^42^

^a^MMP: matrix metalloproteinase; PPE: porcine pancreatic elastase; HLE: human leucocyte elastase.

A protein extract was able to reduce the expression of MMP-1 at the mRNA and protein levels was obtained from the microalgae *Chlorella minutissima*^40^. Also, a *Chorella*-derived peptide was found to inhibit UVB-induced expression of MMP-1, in UVB irradiated human fibroblasts, by suppressing the expression of the ECM-associated signalling protein CYR61, the transcription factor AP-1, and the production of the chemotactic factor MCP-1. These results are crucial since the up-regulation of CYR61 triggers alterations of type I collagen similarly to those verified in photoaged and chronologically-aged skin, once UV irradiation induces the transcription of AP-1 and MCP-1, which in turn stimulates MMP-1 expression^41^.

*Arthrospira maxima* is another example of cyanobacteria able to produce anti-collagenase peptides. The peptide fraction PHS showed anti collagenase activity (92.5%) with an IC_50_ of 32.5 µg/mL compared with the synthetic inhibitor (57.13%)^42^. These peptides sequence can resemble the cleavage site in native collagen, and thus prevent the degradation of the ECM. A competition with the enzyme active site was pointed out as the blocking action of the collagenase by these peptides.

#### 3.1.1.2. Gelatinases

Gelatinases (MMPs-2 and −9) degrade basement membrane and denatured structural collagens^30^^,^^36^. These enzymes are essential in digesting collagen fragments after their initial cleavage by collagenases^43^. Although in lower amount, there are also reports on the potential of cyanobacteria-derived compounds to act upon gelatinases (Figure 1, Table 1). Kunte and Desai^44^ evaluated the effect of the phycobiliprotein C-phycocyanin containing protein extract (C-PC extract) obtained from the cyanobacteria *Spirulina platensis,* in the human gelatinases MMP-2 and MMP-9. The authors found that, besides significantly reducing the activity of MMP-2 by 55.13% and of MMP-9 by 57.9%, C-PC extract also reduced the mRNA expression of both gelatinases, in the hepatocellular cancer cell line HepG2. Although the exacts’ mechanism of inhibition remains unknown, these findings can lead to newer insights for *S. platensis* as a potential source of therapeutically bioactive molecules. A year later, the same authors found another protein extract from *Chlorella minutissima* that successfully reduced the mRNA expression of human MMP-2 and 9, and also upregulated mRNA expression of the tissue inhibitor of metalloproteinases-3 (TIMP-3)^40^.

#### 3.1.1.3. Elastase

Elastase (MMPs-12) is a serine protease with unique ability to digest elastin. After collagen, elastin is the most abundant constituent of the connective tissue in the dermis^4^^,^^37^. The degradation of elastin fibres results in the loss of skin elasticity and, consequently, in a sagging and aged appearance. MMP-12 is the most effective MMP against elastin, and it is produced by macrophages and fibroblasts in response to UV radiation^45^. Several recent reports addressed the ability of natural compounds derived from cyanobacteria and microalgae to overcome elastase overactivity (Figure 1, Table 1). It was recently found that the cyclic depsipeptides tutuilamides A − C, isolated from the cyanobacteria *Schizothrix* sp. and *Coleofasciculus* sp., act as potent inhibitors of porcine pancreatic elastase (PPE), through a reversible binding mode similar to those of the natural cyanobacteria compound lyngbyastatin^46^. Following the National Cancer Institute parameters, we can consider that tutuilamides A − C presented incredible low IC_50_ values (1.18 nM, 2.05 nM and 4.93 nM), being tutuilamide A (Figure 2) the most effective cyanobacteria-derived compound regarding elastase inhibition. Structural analysis of tutuilamide A complexed with PPE confirmed an additional hydrogen bond between the 4-chloro-3-methylbut-3-enoic acid residue and the backbone amide group of elastase residue R226, that appears to stabilise the ligand and may explain the increased inhibitory potency of the compound. In fact, tutuilamide A showed a higher elastase inhibition potential when compared to other compounds such as lyngbyastatin 7, where this additional interaction does not occur^46^.

Other compounds such as the cyclic depsipeptides lyngbyastatin*-*4, -5, -6, and -7,somamide B, tiglicamides A-C, and largamides A-C, produced by *Lyngbya* spp*.,*were shown to selectively inhibit PPE *in vitro*^47–49^. Lyngbyastatin -5, -6, -7, and somamide B inhibited elastase in a competitive way, following the Michaelis-Menten kinetics. The 2-amino-2-butenoic acid (Abu) moiety of the hexadepsipeptide core appears to be the main contributor to the selectivity for elastase^48^. The activity of largamides A-C and tiglicamides A-C in elastase inhibition was inferior to lyngbyastatin 4–7^49^^,^^50^. Later, three new members of lyngbyastatins, namely lyngbyastatins 8, 9 and 10 isolated from the marine cyanobacteria *Lyngbia semiplena*, were also found to inhibited PPE, with IC_50_ values ranging from 120 to 210 nM^51^. Even though these are high IC_50_ values, they denote the potentiality of lyngbyastatins for elastase inhibition, and open doors for further studies where chemical modifications may be considered to increase the compounds activity and specificity. Of the lyngbyastatins evaluated so far, lyngbyastatin 5 (Figure 2) and lyngbyastatin 6 were the most effective against elastase, with IC_50_ values of 3.2 and 3.3 nM, respectively. Within the same genus, Rubio and his team^52^ isolated two cyclic depsipeptides analogues of dolastatin 13, bouillomides A and B, from the cyanobacteria *Lyngbya bouillonii*, and found their capacity to selectively inhibit these serine proteases, although with a higher IC_50_ (1.9 µM). The Abu moiety is also present in these two compounds, which reinforces its role in the selectivity for elastase. Another Abu moiety-containing cyclic depsipeptide, stigonemapeptin, isolated from *Stigonema* sp., also showed selective elastase inhibitory activity, with an IC_50_ of 0.26 µM^53^.

Salvador and co-workers^54^ demonstrated that symplostatin 5–10 (Abu containing cyclic depsipeptides) (Figure 2), isolated from the cyanobacteria *Symploca* sp., potently inhibited the proteolytic activity of elastase (IC_50_ of 37 to 89 nM), which was comparable to the activity of the related compounds lyngbyastatin 4 and 7. It was also shown that compounds containing N-Me-Tyr (symplostatin 8–10) were slightly more potent than their N-Me-Phe (symplostatin 5–7) congeners in inhibiting PPE elastase and human neutrophil elastase. These compounds, with high specificity for elastase, attenuated the effects of elastase in receptor activation, exhibited a superior activity than the clinically approved elastase inhibitor sivelestat, in short-term assays, and also demonstrated superior sustained activity in longer term assays^54^.

The cyclic depsipeptides oscillapeptins A, B, D, and E, isolated from *Oscillatoria agardhii,* inhibited elastase with IC_50_ values of 0.3, 0.05, 30, and 3.0 µg/mL, respectively. The structure/activity analysis of these compounds suggested that the presence of an amino acid residue between Thr and the 3-amino-6- hydroxy-2-piperidone (Ahp) unit is essential in the selectivity^55^. Tricyclic peptide microviridin I showed inhibitory activity on elastase with an IC_50_ of 0.34 µg/mL. The cyclic depsipetides containing the Ahp moiety such as oscillapeptin G and oscillapeptilides 97-A, -B, were also recognised as elastase inhibitors (IC_50_ =0.73, 0.42 and 1.12 µg/mL)^56^. Other microviridin-type peptides (G and H) and nostopeptins (A and B), produced by *Nostoc minutum,* have also demonstrated ability to prevent elastin degradation through elastase inhibition^57^^,^^58^. The cyanobacteria *Microcystis aeruginosa* was also shown to produce microviridins, namely microviridins B and C, which inhibited elastas with IC_50_ = 0.044 and 0.084 µg/mL, and micropeptins HH978, HH960, HH992, and DR1006, withIC_50_ = 17.6, 55.5, 16.9 and 13.0 µM, respectively. Microviridins B and C had similar IC_50_ against elastase as G and H, and this observation can be explained, at least in part, by the molecular structure: it was reported that the amino acid sequence of X-Thr-Y affects elastase inhibitory activity, and both microviridins B, C, G, and H presented a hydrophilic amino acid residue in the place of X, and a Leu in the place of Y^59–61^.

A new peptide, molassamide, from *Dichothrix utahensis,* was found to have serine protease inhibitory activity against elastase with IC_50_ = 0.032 µM, and a similar selectivity profile as those previously described for lyngbyastatin 4–7, maybe due to their structural similarity^62^. Two other cyclic depsipeptides with activity against elastase were isolated from *Scytonema hofmanni*, and designated scyptolin A and B^63^. A correlation between the molecular structure and the bioactivity can also be predicted, once two distinguish features were observed: the fifth position replaced by Leu, as previously reported in microviridins, and a 3-chloronated N-methyl-Tyr residue in eighth position. As in the previous studies, PPE was used as model, and scyptolin A and B were reported to block elastase activity at low concentrations. It has been shown that scyptolins bind directly into the active centre of the target peptidase, in a substrate-like manner, however, the molecular basis of this selectivity is still unclear^63^.

*Planktothrix rubescens* is another cyanobacteria that produces elastase inhibitors (planktopeptin BL1125, BL843, and BL106) with IC_50_ values of 96 nM, 1.7 µM, and 40 nM, respectively. After the examination of the molecular structure of these compounds, it was possible to predict a structure-activity relationship, being revealed that the flexible side chain of the molecules showed marginal selectivity for elastase. BL1125 is a liner competitive tight-binding inhibitor of human leukocyte (HLE) (K_i_ =2.9 nM) and pancreatic (K_i_ =7.2 nM) elastase, and is effective in inhibiting the cleavage, not only of the synthetic substrate, but also of elastin of natural provenance. HLE has become more relevant due to its involvement in several pathological processes so, finding inhibitors for this enzyme has a strategic therapeutic interest^64^. Years later, Bubik and co-workers^65^, discovered the peptides anabaenopeptins B and F from the same cyanobacteria strain, with also the ability to inhibit HLE and PPE, although in a lesser extent than PPBL1125. The inhibition profiles of HLE showed competitive inhibition, with the K_i_ values between 0.1–1 µM. Regarding PPE, the profiles revealed a sigmoid shape, which describes the binding of two inhibitor molecules to the enzyme. The first inhibitor molecule had a K_i_ ranging from 1–2 µM and, the second, presented K_i_ values approximately 50-fold higher^65^.

Inhibition of HLE was also achieved with brunsvicamides A-C, produce by *Tychonema* sp. These compounds were highly selective for HLE with K_i_ values of 1.1, 0.70, and 1.6 µM, respectively, calculated assuming competitive inhibition. It was also reported that brunsvicamides may act as alternate HLE substrates with a strongly decelerated diacylation^66^. Another HLE inhibition was found with the *Nostoc insulare* cyanopeptolins, insulapeptolides A-H. Insulapeptolides A-D had IC_50_ values between 85 (K_i_ value of 36 nM) and 140 nM, hence being highly potent inhibitors, whereas insulapeptolides E-H were less active, with IC_50_ values varying between 1.6 and 3.5 µM. Therefore, it can be concluded that these compounds occupy the substrate-binding site of HLE, suggesting that the insulapeptolides act as competitive inhibitors by gorming non-covalent enzyme-inhibitor complexes with HLE^67^ (Table 1).

The cyclic depsipeptides isolated from cyanobacteria have revealed a huge potential to avoid and slow down elastin degradation through both direct elastase inhibition and interference at the level of enzyme expression. In some situations, the high specificity for the enzyme put these compounds at the forefront for the development of effective and innovative anti-ageing formulations, with potential to maintain and improve dermal filling, and delay the establishment of wrinkles. Of the molecules presented before, tutuilamides and lyngbyastatins seem worthy of further studies by the pharmaceutical and cosmetic industries, in view of their proved potency against this enzyme.

#### 3.1.1.4. Hyaluronidases

Excessive superficial water lost by evaporation greatly contributes to skin ageing. Evaporated water is replaced with water from the innermost epidermal layers and dermis, leading to cell shrinkage, and in a worst scenario to cell death^68^^,^^69^. The relationship between skin hydration and occurrence of wrinkles, demonstrated that skin hydration significantly reduce the depth of wrinkle furrows^70^.

An adequate skin moisture is, in part, achieved through the preservation of hyaluronic acid (HA), due its unique capacity of retaining water. Hyaluronidases (HASEs) are enzymes that break-down polymers by cleaving high molecular weight HA into smaller fragments^4^. HA is the key molecule involved in skin moisture^71^. Its function is, among others, to bind water and to lubricate movable parts of the body^72^. As already stated, HA is degraded into fragments of varying sizes by HASEs^73^. HA is found in young skin at the periphery of collagen and elastin fibres. Aged skin, which is less plump than youthful skin, is characterised by decreased levels of HA. The decrease in HA levels may be involved in the changes noted in aged skin, including wrinkling, altered elasticity, and reduced turgidity^74^. Beating the enzymes responsible for HA degradation seems then an effective strategy to delay skin-ageing and improve the appearance of the skin at all ages.

Regarding HASE inhibitory activity, Yamaguchi and Koketsu^75^ found that the cyanobacteria *Nostochopsis lobatus* MAC0804NAN produce a large amount of a polysaccharide with a high inhibitory effect (IC_50_= 7.18 µg/mL) on HASE, being about 14.5 times stronger than the natural inhibitor disodium cromoglycate. Being an edible species, upholds its use for cosmetic purposes, as well as its acceptance by consumers, since it already has a known safety profile. Besides pure compounds, it was also noticed that extracts, namely ethanol-insoluble fractions, could inhibit the activity of HASE, as shown by Fujitani et al. in a study conducted with seven different genera of microalgae^76^ (Table 1). The IC_50_ of *Spirulina platensis, Porphyridium purpureum, Rhodosorus marinus, Chlorella pyrenoidosa, Dunaliella salina, and Pleurochrysis carterae* was 0.15, 0.18, 0.26, 0.94, 0.15 and 0.41 mg/mL, respectively, with *S. platensis* and *D. salina* presenting similar values to those of the natural HASE inhibitor. It was reported that the ethanol-insoluble fraction included macromolecules such as polysaccharides, which may be involved in hyaluronidase inhibition.

The use of effective extracts as active ingredients for cosmetics production can constitute an asset face to isolated compounds, due to the higher extraction yield and lower processing costs.

### 3.2. Hyperpigmentation

Skin-whitening, as well as an aesthetically pleasing and uniform skin pigmentation, has been a primary focus of many cosmetic industries. Skin often gets irregularly darkened because of the UV radiation, ageing, and pregnancy. Although hyperpigmentation is not harmful in any way, sometimes it may cause serious problems, such as melanoma. As a result, several treatment modalities are being investigated for their efficacy to treat skin pigmentation disorders^10^^,^^77^.

Melanogenesis occurs in melanocytes, located at the base of epidermis, in a process involving several chemical and enzymatic reactions to produce melanin, a major component of skin colour^78^. Hyperpigmentation can occur through an increase in the number of melanocytes, or through the overactivity of melanogenic enzymes - Tyrosinase^79^^,^^80^ (Figure 1). The accumulation of abnormal amount of melanin is mainly caused by UVR exposure, which increases reactive oxygen species (ROS) production^78^. ROS are produced in the epidermis of the skin and stimulate melanocytes to convert tyrosine into melanin by oxidation, through the action of tyrosinase^10^. Tyrosinase is a crucial enzyme that catalyses melanin synthesis in melanocytes. Therefore, skin pigmentation can be prevented by tyrosinase inhibitors^10^^,^^81^. Some of the well-known tyrosinase inhibitors are hydroquinone (HQ), kojic acid, and arbutin. Although being effective as depigmenting agents, these compounds are not devoid of harmful effects. It has already been demonstrated that HQ has mutagenic effects and cytotoxicity against mammalian V79 cells^82^, causes DNA damage^83^ and has some evidence of carcinogenic activity^84^. Regarding kojic acid, skin irritation and allergic dermatitis were developed after using skincare products containing it^85^. In relation to arbutin, the application of higher concentrations caused skin irritation and hyperpigmentation^86^. Additionally, they have high toxicity, low stability, poor skin penetration, and insufficient activity^87^. Face to the exposed, it is extremely important to find alternatives to overcome hyperpigmentation, or to find new tyrosinase inhibitors with effectivity and less harmful side effects. The research on tyrosinase inhibition by cyanobacteria and microalgae-derived compounds has been very limited to date, and the majority of the available studies explore mushroom tyrosinase as enzymatic model, making it difficult to translate the results to human environment. However, some promising compounds and bioactive extracts from cyanobacteria and microalgae have emerged in the last years (Table 2).

**Table 2.** Bioactive potential of cyanobacteria and microalgae-derived extracts and isolated compounds in skin-whitening.^a^

Compound/extract	Species	Model	Reference
Ethanol and water extracts	*Arthrospira platensis*	Mushroom tyrosinase	Sahin^87^
Oscillapeptin G	*Oscillatoria agardhii*	Mushroom tyrosinase	Sano and Kaya^88^
C-phycocyanin Vitamins C and E	*Spirulina* sp.	B16F10 murine melanoma cells -	Wu et al.^89^ Babadzhanov et al.^95^
PHORMISKIN Bioprotech G^®^	*Phormidium persicinum*	Humans	Phormiskin bioprotech g^91^
Mono and Diesters of Astaxanthin	*Haematococcus pluvialis*	Mushroom tyrosinase	Rao et al.^92^
Zeaxanthin in submicronsized precipitates	*Nannochloropsis oculata*	Mushroom tyrosinase	Shen et al.^93^
Purified peptide	*Pavlova lutheri*	Hydroxyl radical scavenging activity ABTS B16F10 murine melanoma cells	Oh et al.^90^
Vitamins C and E	*Chlorella vulgaris*	Muscle and hepatopancreas of the *M. rosenbergii*	Radhakrishnan et al.^96^

^a^ABTS: 2,2’-azino bis (3-ethylbenzothiazoline-6-sulfonic acid) as substrate in a colorimetry assay.

Examples of the potential of cyanobacteria in hyperpigmentation include crude extracts of *Arthrospira platensis*. Sahin^87^, found that ethanol and water extracts of *A. platensis* presented IC_50_ values in a comparable scale to those of kojic acid. It was found that some phenolic compounds produced by this species, e.g. caffeic and ferulic acids, and present in the extracts, are considered to be the most effective inhibitors of the enzyme tyrosinase, with IC_50_ values significantly lower than those of the drugs kojic acid and arbutin. Although the authors have undertaken the study in a non-human enzyme model, the comparison of their results with those of the human tyrosinase inhibitors, points *A. platensis* extracts as alternative precursors in obtaining both effective and safer inhibitors for tyrosinase activity^87^.

In 1996, a study performed with *Oscillatoria agardhii* demonstrated that oscillapeptin G exhibited tyrosinase inhibition in 55% face to the untreated control, but no mechanistic or reference drugs were explored^88^. A more complex research in this thematic has been undertaken by Wu and co-workers, who explored the anti-melanogenic effect of C-PC from *Spirulina* sp., using B16F10 murine melanoma cells. The authors found that C-PC inhibits melanin biosynthesis by a dual mechanism, one promoting the degradation of MITF protein, the transcription factor of tyrosinase, through the up-regulation of MAPK/ERK signalling pathway, and the other by suppressing the activation of CREB, the transcription factor of MITF, via the down-regulation of p38 MAPK pathway^89^. Oh and co-workers^90^ explored a novel peptide isolated from *Pavlova lutheri* in ROS generation and expression of melanogenic specific proteins. The authors found that the peptide demonstrated inhibitory properties against α-Melanocyte Stimulating Hormone-induced melanogenesis via melanin content, tyrosinase inhibition in B16F10 melanoma cells, and also decreased melanogenesis-related proteins. Therefore, this protein has potential whitening effects and prominent protective effects on oxidative stress-induced cell damage, which can be used as an effective natural source in cosmeceutical and pharmaceutical products.

Despite the scarce *in vitro* trials in the thematic of hyperpigmentation using cyanobacteria and microalgae-derived compounds, the company CODIF Research & Nature took a step forward with a trial involving human volunteers. This biotechnological company, that explores sea resources for cosmetics production, developed a biotechnological extract, PHORMISKIN Bioprotech G®, from the cyanobacteria *Phormidium persicinum,* able to reduce melanin synthesis. In a study, 15 volunteers aged between 25 and 46 years old applied the extract, in a concentration of 2%, during 28 consecutive days. After the experimental period, the skin tone became more uniform and the skin brighter. The extract also stimulated the synthesis of the protein thioredoxin, which is known for its antioxidant and detoxifying properties^91^.

There are also some studies with bioactive extracts and compounds isolated from microalgae. One of them uses astaxanthin from *Haematococcus pluvialis,* and shows the multitarget action of this xanthophyll, namely in the inhibition of ROS accumulation and down-regulation of tyrosinase. In this study, astaxanthin diesters had the highest tyrosinase inhibitory activity than monoesters, presenting IC_50_ values of 2.12 and 3.5 µg/mL, respectively. Hence the mentioned properties may prevent the uncontrolled proliferation and accumulation of melanocytes, and consequently of melanin^92^. Also, other survey with zeaxanthin from *Nannochloropsis oculata* reported tyrosinase inhibitory activity in a dose-dependent manner^93^, assuming the potential of xanthophylls as brightening agents.

Another approach to prevent melanosome formation in the skin is by using vitamins C and E^94^. In this regard, it can be assumed that *Spirulina* sp.^89^^,^^95^ and *Chlorella vulgaris*^96^ constitute great candidates for cosmetic purposes, due to their significant content in these vitamins.

In an attempt to point a possible structure-activity relationship, and taking into account the IC_50_ values found for the different cyanobacteria and microalgae-derived compounds, it seems that phenolic acids like caffeic and ferulic acid, present in bioactive extracts, and with a molecular structure more similar to kojic acid, are more effective in inhibiting tyrosinase than peptides.

## 4. Future perspectives

The concern in delaying the effects of ageing has been the fuel for the investment in the search for new, innovative, effective and eco-friendly compounds, aiming the discovery of the perfect anti-ageing formulation. Allied to this, the growing research in natural sources, more specifically from marine origin, has provided a countless number of new molecules with promising bioactivities worth of further exploitation in the field of skin ageing. Besides the inherent advantages of using cyanobacteria and microalgae as metabolic producers, it has been demonstrated herein their huge potential to target specific enzymes involved in the ageing process, most of the times with an activity significantly higher than that of the reference drugs currently in use. In this regard, it seems unquestionable that further toxicological and biotechnological studies will be the next steps to evaluate the safety and effectiveness of these molecules in anti-ageing formulations, and that they will much probably revolutionise the cosmetic market.
